# Does interprofessional team-training affect nurses’ and physicians’ perceptions of safety culture and communication practices? Results of a pre-post survey study

**DOI:** 10.1186/s12913-021-06137-5

**Published:** 2021-04-14

**Authors:** Jan Schmidt, Nikoloz Gambashidze, Tanja Manser, Tim Güß, Michael Klatthaar, Frank Neugebauer, Antje Hammer

**Affiliations:** 1 Institute for Patient Safety, University Hospital of Bonn, Venusberg-Campus 1, 53127 Bonn, Germany; 2 FHNW School of Applied Psychology, University of Applied Sciences and Arts Northwestern Switzerland, Riggenbachstrasse 16, 4600 Olten, Switzerland; 3 UKM Trainingszentrum, University Hospital of Muenster, Malmedyweg 17, 48149 Muenster, Germany; 4 QM and clinical risk management, University Hospital of Muenster, Domagkstrasse 20, 48149 Muenster, Germany

**Keywords:** Patient safety, Interprofessional team training in hospitals, Implementation, Communication in health care, Safety culture

## Abstract

**Background:**

Many hospitals seek to increase patient safety through interprofessional team-trainings. Accordingly, these trainings aim to strengthen important key aspects such as safety culture and communication. This study was designed to investigate if an interprofessional team-training, administered to a relatively small group of nurses and physicians would promote a change in healthcare professionals’ perceptions on safety culture and communication practices throughout the hospital. We further sought to understand which safety culture aspects foster the transfer of trained communication practices into clinical practice.

**Methods:**

We conducted a pre-post survey study using six scales to measure participants’ perceptions of safety culture and communication practices. Mean values were compared according to profession and participation in training. Using multiple regression models, the relationship between safety culture and communication practices was determined.

**Results:**

Before and after the training, we found high mean values for all scales. A significant, positive effect was found for the communication practices of the physicians. Participation in the training sessions played a variably relevant role in the communication practices. In addition, the multiple regression analyses showed that specific safety culture aspects have a cross-professional influence on communication practices in the hospital.

**Conclusions:**

This study suggest that interprofessional team-trainings of a small group of professionals can successfully be transferred into clinical practice and indicates the importance of safety culture aspects for such transfer processes. Thus, we recommend the consideration of safety culture aspects before starting a training intervention.

**Supplementary Information:**

The online version contains supplementary material available at 10.1186/s12913-021-06137-5.

## Background

Effective collaboration and communication in interprofessional teams are key to high quality and safety in healthcare delivery. Studies have shown that poor team communication contributes to potentially avoidable adverse events and patient harm [1–4]. Moreover, complex patient care involves clinicians from multiple specialties and professional backgrounds and requires frequent handovers and transitions. Thus, skills in interprofessional team communication are fundamental to ensuring effective information transmission along the patient care process

Communication practices such as 2-way-communication (closed-loop communication), briefings, and feedback can support interprofessional communication and hence contribute to improved quality and safety of care [5–7]. 2-way-communication is a communication technique in which a received verbal message is followed by an explicit confirmation to the sender of the message. This method is already used in high-risk sectors, such as the army and aviation, to avoid misunderstandings and to confirm actions taken. Briefings are used in interprofessional teams to create an equal level of information, discover unsolved problems, and establish or maintain a common understanding of the situation. This should minimize the risk of possible loss of information [8]. Feedback is used to reflect on the performance of the team as well as the individual performance, and can lead to alternative solutions in the future, or strengthen existing good practices.

Team-trainings were shown to be effective at improving communication processes in healthcare [9, 10], especially if following a holistic, organisation-wide and interprofessional approach [11]. However, such approaches are challenging to implement and depend heavily on organisational culture [12–14]. Different professions in the hospital setting usually have different education and qualifications, possess different roles and use different professional jargons, all of which may lead to different perspectives on patient safety [15, 16]. Consequently, small-scale local trainings of communication skills, especially delivered for individual professional groups, may have limited impact on interprofessional collaboration..

In 2015, the University Hospital Muenster launched an interdepartmental, interprofessional training project to strengthen safety culture and train employees in communication skills. The project group, ‘Safety Training’, therefore developed interprofessional team-training courses for relatively small groups of nurses and physicians (9% of overall participants), representing 17 participating departments with a total of approximately 2000 employees. These representatives of nurses and physicians from participating departments served as so-called ‘champions’ to transfer training contents into clinical practice [17]. However, it is unclear how many trained champions are required to initiate change at the department level and which cultural aspects support a transfer of training content into clinical practice.

In patient safety research, a culture of safety is generally considered an important factor for improving healthcare delivery [18–21]. Safety culture is a multidimensional construct [22], previous studies identified different safety culture aspects as important facilitators for successful implementation of quality improvement initiatives. Leadership [23, 24], teamwork [25, 26], and psychological safety [27] were identified as strong catalysts to successfully implement quality improvement strategies such as interprofessional trainings. Thus, we seek to understand whether these aspects of safety culture support the interdisciplinary training of patient safety champions in the hospital setting.

## Research questions/objectives

Firstly, this study aims to investigate, if the interprofessional team-training of champions can be successfully transferred into clinical practice. Thus, we examine whether there are changes in professionals’ perceptions on safety culture aspects and communication practices before and after the intervention and whether the results differ between training participants and nonparticipants.

Second, we seek to understand which safety culture aspects serve to foster the transfer of trained communication practices into clinical practice. In this regard, we seek to understand the relevance of nurses’ and physicians’ perceptions on safety culture aspects and its influence on trained communication practices. Results from this study will help to understand if trained champions can make a difference at the department level and what cultural aspects are instrumental in making that happen.

## Methods

### Study context

Between January and November 2016, the project group conducted a series of interprofessional team-trainings of clinical managers and champions. The University Hospital Muenster comprises about 9600 employees working in 42 departments. Of these, 17 departments (with approximately 2000 employees) participated in team-trainings. These departments were chosen based on either their high patient flow, their risky profile for patient care or their time-critical processes and/or complex interprofessional composition (e.g. operating areas, intensive care units, and emergency outpatient departments).

### Training concept and implementation

**Fig. 1:**
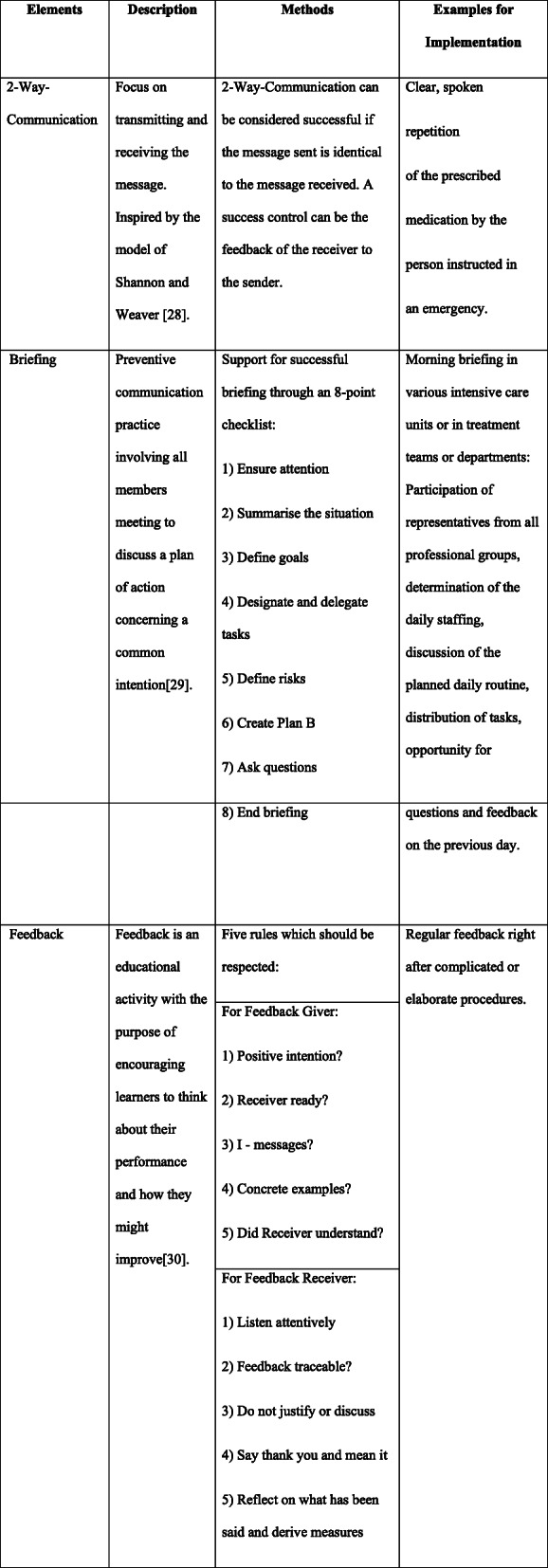
Description of the key aspects of communication and clarification using methods and examples of the study

The interprofessional team-trainings aimed to increase employees’ awareness of safety culture within the organisation [27] and to improve the use of standardised communications practices (i.e. 2-way-communication, briefing, and feedback). Descriptions and examples are shown in Fig. 1 [28–30].

The trainings have been conducted at both the (1) clinical management level and (2) frontline professional level. Clinical managers are responsible for translating senior executives’ visions into routine practices, facilitating efforts in specific improvement strategies, promoting innovative practices, and supporting frontline professionals’ activities for these strategies [31]. Concurrently, this provides an opportunity for transferring frontline needs and information upwards, thus drawing senior executives’ attention to specific requirements at local level [31]. Because frontline professionals are familiar with local requirements for implementing selected communication tools, they served as champions for actions taken in respective departments. Thus, the trainings compiled management trainings for 108 physicians and nurses with executive functions and champion trainings for 71 frontline professionals, reaching a total of 9% of staff in participating departments.

Trainings were conducted in two modules covering the communication practices and providing a set of communication tools that could support local implementations of actions. The first training module (two days) included communication practices and communication tools to support local implementation (e.g. structured briefings, check-backs, avoidance of ‘killer’ phrases). In the second module, training participants were introduced to the concept of safety culture and invited to reflect on implementation progress and share experiences across departments.

**Fig. 2:**
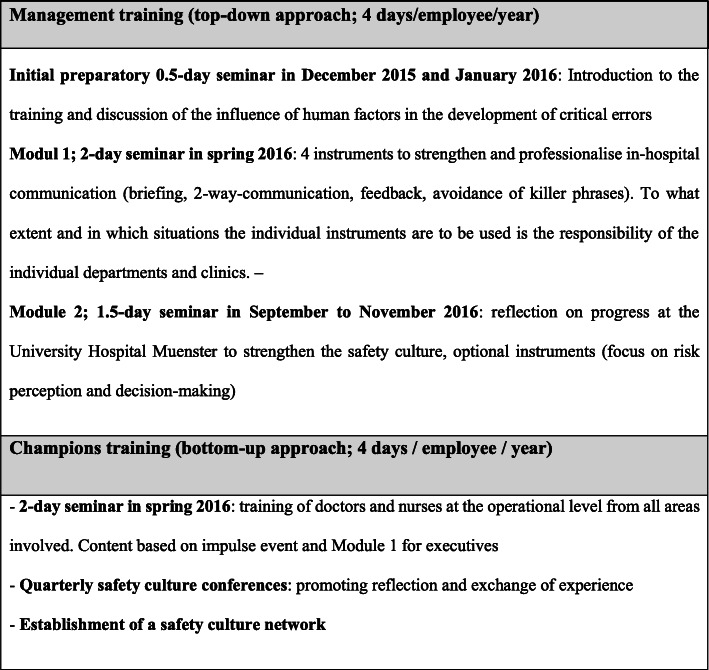
Overview and content of trainings

As part of an organisational learning process, the latter phase enabled participants – especially the champions – to learn from each other on successful local strategies for transferring training contents into local practice. The trained champions were introduced to possible ways of transferring training contents into practices (e.g. small team trainings, weekly briefings or introduction of checklists). Based on the local needs and context, they could decide how to pass on the contents of the trainings. In the second half of the study phase, the project management initiated additional complementary actions at local level (e.g. feedback workshops, observations of handovers, supervisions) in order, to assess the current status, identify problems and support the transfer from training to practice. An overview of all trainings is displayed in Fig. 2.

### Data collection procedure

To evaluate the impact of the interprofessional team-training on the perception of safety culture and communication practices, the Institute of Patient Safety conducted a pre-post online survey of all employees in participating departments. In January and February 2016, before the first training module, approximately 2000 clinicians were invited to participate in a baseline survey (t_0_) – including the 179 participants of the training intervention. The second survey (t_1_) was conducted six months after completion (of the second training module in September to November 2016). All participants were invited via email and received reminders two and four weeks after the initial invitation. Participation was voluntary and due to data security aspects, survey links at t_0_ and t_1_ were sent independently to all employees to enter data anonymously. Thus, we were not able to link participants in pre- and post-surveys nor to request information on non-responders.

### Measures

The safety culture aspects *Supervisor Expectations* (four items, Cronbach’s alpha 0.75) and *Teamwork Within Units* (four items, Cronbach’s alpha 0.77) were measured with two scales from the German version of the Hospital Survey on Patient Safety Culture (HSPSC) [32, 33]. Answers for these two scales were given on a 5-point Likert scale (1 = strongly disagree to 5 = strongly agree). *Psychological Safety* (seven items Cronbach’s alpha 0.84) was measured using the German adaptation from Edmondson [34, 35]. Answers were given on a 5-point Likert scale (1 = not true at all to 5 = absolutely true).

In order to measure communication practices, we developed three scales capturing the main communication practices covered in the trainings: *2-Way-Communication* (three items, Cronbach’s alpha = 0.88), *Briefing* (three items, Cronbach’s alpha = 0.78) and *Feedback* (five items, Cronbach’s alpha = 0.83). Answers were given on a 5-point Likert scale (1 = not true at all to 5 = absolutely true).

To cover information on sociodemographic characteristics, participants were asked about their profession (1 = nurse, 2 = physician, 3 = other), leadership position (0 = no, 1 = yes), and their participation in the interprofessional team-training (0 = no, 1 = yes).

An overview of scales and items used in the analysis is provided in an additional table (Additional Table 1). The entire German pre-post survey is available on request by contacting the last author.

### Statistical analyses

Prior to analyses, cases with more than 30% missing in survey items were excluded from the data set to ensure sufficient data quality. Negatively-worded items were reverse coded for further analyses.

In pre-analyses, we calculated frequencies on participants’ profession, leadership position and participation in interprofessional team-training. Other professional groups besides nurses and physicians were not considered in the further calculations due to their limited numbers. We calculated descriptive statistics (means and standard deviations (SD)) for all six scales (three for safety culture and three for communication) in pre- and post-measures separately for nurses and physicians. In order to identify difference in perceptions of trained and non-trained professionals, we additionally calculated descriptive statistics and used Mann-Whitney-U-tests to analyse changes of means from t_0_ to t_1_ of these six scales for training participants and nonparticipants separately for nurses and physicians, by solely using answers given at t_1._ Significance level was set at *p* < 0.05. The Cohen-test was used to determine the strength of the significant results in Mann-Whitney-U-test. Results 0.2 ≤ r < 0.5 were considered weak, from 0.5 ≤ r < 0.8 medium and r > 0.8 strong [36].

In order to identify relationships between safety culture aspects and communication practices, we used the Spearman test. Analyses were conducted separately for nurses and physicians.

Finally, we investigated which specific perceptions of safety culture aspects influence the perceptions on communication practices in nurses as well as physicians by running stepwise multiple regression analyses per each of the three communication practices (2-Way-Communication, Briefing and Feedback) as dependent variable and safety culture aspects as independent variable. Regression models were conducted separately for nurses and physicians and points in time. We used multiple linear regression with backwards selection and set the significance level at 5% (*p* < 0.05). We calculated regression coefficient (β), explained variance (R^2^), and the corresponding effect size (f^2^) for each model. Effects 0.02 ≤ f^2^ < 0.15 were considered small, 0.15 ≤ f^2^ < 0.35 medium and f^2^ ≥ 0.35 strong [37]. All analyses were performed with IBM SPSS Statistics V.25.

## Results

Of 2038 and 2045 employees invited in 2016 (t_0_) and in 2017 (t_1_), 569 (27.92%) and 402 (19.66%) participated in the online survey. After removing cases with more than 30% missing items, 528 (t_0_) and 366 (t_1_) cases were included in further analyses. Of these cases, 30.30% were physicians at t_0_ (25.68% at t_1_) and 58.90% nurses at t_0_ (63.11% at t_1_). At both measurement points, about a quarter of the participants indicated having leadership functions. At t_1_, after completion of trainings, the percentage of respondents who stated that they participated in trainings was 33.61% Table 1 provides an overview of participant characteristics at t_0_ and t_1_.

**Table 1:** Participant characteristics in t_0_ (2016) and t_1_ (2017)

	t_**0**_ (2016)	t_**1**_ (2017)
	N (%)	N (%)
**Participants of the study**		
Number of employees at the departments	2038	2045
Total of participants	569 (27.92)	402 (19.66)
Total of participants after excluding Missing > 30%	**528** (25.91)	**366** (17.90)
**Profession**		
Nurses	311 (58.90)	231 (63.11)
Physicians	160 (30.30)	94 (25.68)
Others	57 (10.80)	41 (11.20)
Missing	0 (0.00)	0 (0.00)
**Leadership position**		
Yes	134 (25.38)	88 (24.04)
No	391 (74.05)	274 (74.86)
Missing	3 (0.57)	4 (1.09)
**Participation in training**		
Yes		123 (33.61)
No		242 (66.12)
Missing		1 (0.27)

### Changes in nurses’ and physicians’ perceptions on safety culture aspects and communication practices

Means and standard deviations of all safety culture and communication scales at t_0_ and t_1_, and Mann-Whitney-U-tests are presented separately for nurses and physicians in Table 2. Overall, t_0_ results showed relatively high values for both nurses and physicians in the three safety culture aspects *Supervisor Expectations*, *Teamwork Within Units* and *Psychological Safety*, with physicians generally rating all three scales more positively compared to nurses. At t_1_, we found a slight decrease in mean values for *Supervisor Expectations* and *Teamwork Within Units* for both professions. For *Psychological Safety,* we observed a decrease for physicians while nurses reported slightly more positive perceptions of *Psychological Safety* at t_1_. However, none of these differences were significant.

**Table 2:** Safety culture aspects and communication scales by professions and study periods/ training participation

Profession	Nurses			Physicians		
**Study periods** **t**_**0**_ **(2016)/ t**_**1**_**(2017)**	t_0_ (2016) Mean (SD)	t_1_(2017) Mean (SD)	Δ	t_0_ (2016) Mean (SD)	t_1_(2017) Mean (SD)	Δ
Safety Culture Aspects						
Supervisor Expectations	3.42 (0.72)	3.38 (0.71)	−0.04	3.58 (0.72)	3.47 (0.78)	−0.11
Teamwork Within Units	3.42 (0.60)	3.36 (0.61)	−0.06	3.64 (0.69)	3.54 (0.78)	−0.10
Psychological Safety	3.61 (0.66)	3.69 (0.61)	0.08	3.68 (0.67)	3.67 (0.66)	−0.01
Communication Practices						
2-Way-Communication	3.69 (0.94)	3.65 (0.91)	−0.04	3.19 (0.96)	3.52 (0.84)	0.33**
Briefing	3.15 (0.86)	3.27 (0.79)	0.12	3.46 (0.87)	3.74 (0.79)	0.28*
Feedback	2.92 (0.81)	2.86 (0.83)	−0.06	3.07 (0.79)	3.22 (0.79)	0.15
**Training participants/ non-participants** **(at t**_**1**_**)**	non-participants Mean (SD)	participants Mean (SD)	Δ	non-participants Mean (SD)	participants Mean (SD)	Δ
Safety Culture Aspects						
Supervisor Expectations	3.36 (0.72)	3.44 (0.71)	0.08	3.34 (0.81)	3.70 (0.70)	0.36
Teamwork Within Units	3.33 (0.62)	3.43 (0.61)	0.10	3.42 (0.84)	3.72 (0.62)	0.30
Psychological Safety	3.64 (0.63)	3.80 (0.55)	0.16	3.52 (0.72)	3.91 (0.46)	0.39**
Communication Practices						
2-Way-Communication	3.70 (0.92)	3.54 (0.90)	−0.16	3.44 (0.90)	3.64 (0.72)	0.20
Briefing	3.26 (0.79)	3.30 (0.81)	0.04	3.54 (0.83)	4.07 (0.58)	0.53**
Feedback	2.83 (0.85)	2.92 (0.78)	0.09	3.09 (0.84)	3.46 (0.65)	0.37*

Notes: Means, standard deviations (SD) and deltas (Δ) for all six scales of safety culture aspects and communication practices regarding points in time and training participation. Mann-Whitney-U-test significance: *p < 0.05 ** *p* < 0.01

Concerning communication practices, nurses rated *2-Way-Communication* in the t_0_-survey, higher than physicians. The values of *2-Way-Communication* for nurses decreased slightly in the post evaluation (t_1_). By comparison, the mean value of *2-Way-Communication* for physicians increased clearly from t_0_ to t_1_ and approached the mean value of nurses at t_1_. The Mann-Whitney-U-test confirmed a significant difference in pre-post evaluations of *2-Way-Communication* by physicians (U = 5598.50 *p* < 0.01); (Table 2). The effect size according to Cohen was r = 0.2. The mean values of *Briefing* showed high values for both professions in both points in time and the values were more positive for nurses and physicians at t_1_ than at t_0_. However, only the result for physicians reached statistical significance (U = 6177.60, *p* = 0.02). The effect size, according to Cohen, was r = 0.15. *Feedback* showed lowest values for both professions and at both measurements. At t_1_, nurses’ perceptions resulted in lower mean values compared to t_0_, while the physicians’ ratings increased. However, differences were not significant.

Comparing the mean values for training participants and nonparticipants at t_1,_ results showed generally higher mean values in all 6 scales for participating physicians (Table 2). For nurses who had participated in the trainings, mean values were higher on 5 out of the 6 scales (except for *2-Way-Communication*) compared to nurses who had not participated. However, none of these differences reached statistical significance. For physicians, all mean values of training participants were higher than those of nonparticipants. These differences proved to be significant for *Psychological Safety* (U = 673.500, *p* = 0.008), *Briefing* (U = 614.000, *p* = 0.002) and *Feedback* (U = 704.000, *p* = 0.017).

### Relationship between safety culture aspects and communication practices

Results of the correlation analysis are presented in Table 3. We found higher correlations between all aspects of safety culture and communication practices at t_1_ than at t_0_ for physicians. However, we did not identify uniform changes in correlations of nurses’ perceptions at t_1_.

**Table 3:** Correlation between the scales of safety culture aspects and communication practices for both professional groups

**Nurses**	**t0 (2016)**					**t1(2017)**
Variable	1	2	3	4	5	6
1) Supervisor Expectations	–	0.38***	0.44***	0.08	0.31***	0.52***
2) Teamwork Within Units	0.39**	–	0.51***	0.08	0.25***	0.28***
3) Psychological Safety	0.43**	0.56***	–	0.19**	0.35***	0.53***
4) 2-Way-Communication	0.12*	0.19***	0.18**	–	0.41***	0.36***
5) Briefing	0.34***	0.39***	0.35***	0.39***	–	0.55***
6) Feedback	0.40***	0.48***	0.47***	0.28***	0.53***	–
**Physicians**	**t0 (2016)**					**t1 (2017)**
Variable	1	2	3	4	5	6
1) Supervisor Expectations	–	0.50***	0.54***	0.25*	0.51***	0.55***
2) Teamwork Within Units	0.44**	–	0.65***	0.44***	0.54***	0.65***
3) Psychological Safety	0.57***	0.54***	–	0.41***	0.67***	0.61***
4) 2-Way-Communication	0.20*	0.20*	0.19*	–	0.55***	0.50***
5) Briefing	0.44***	0.52***	0.53***	0.40***	–	0.64***
6) Feedback	0.54***	0.52***	0.52***	0.32***	0.67***	–

Notes: Spearman test for linear correlation between Safety culture aspects and Communication practices

Below the diagonal = t_0_, above the diagonal = t_1_; Higher correlation in either t_0_ or t_1_ highlighted in bold; significance level: *p < 0.05, **p < 0.01, ***p < 0.001

### Impact of safety culture aspects on nurses’ and physicians’ perceptions on 2-way-Communiaction, briefing and feedback

Results of multiple regressions analyses are presented in Table 4. 2-Way-Communication: For nurses, we found a significant effect of *Psychological Safety* on *2-Way-Communication* at both measurement points; t_0_ (β = 0.21, *p* < 0.05) and t_1_ (β = 0.23, p < 0.05). The explained variance remained low and decreased from 5% to 2% at t_1_ (p < 0.05 f^2^ = 0.14). Similarly, for physicians we found significant effects of *Psychological Safety* on *2-Way-Communication* at t_0_ (β = 0.33, *p* < 0.01) and *Teamwork Within Units* on *2-Way-Communication* at t_1_ (β = 0.50, *p* < 0.001). The explained variance increased from 5% at t_0_ to 20% at t_1_ corresponding to a strong effect (f^2^ = 0.50).

**Table 4:** Influence of safety culture aspects on communication practices for points in time and professions

**Nurses**						
	**2-Way-Communication**		**Briefing**		**Feedback**	
**Year**	**t**_**0**_ **(2016)**	**t**_**1**_ **(2017)**	**t**_**0**_ **(2016)**	**t**_**1**_ **(2017)**	**t**_**0**_ **(2016)**	**t**_**1**_ **(2017)**
**Variables (β)**						
Supervisor Expectations	–	–	0.23***	0.20*	0.22***	0.42***
Teamwork Within Units	–	–	0.33***	–	0.35***	–
Psychological Safety	0.21*	0.23*	0.24**	0.41***	0.33***	0.53***
**Explained variance R**^**2**^	0.05***	0.02*	0.23***	0.17***	0.34***	0.40***
**N**	309	228	208	227	309	228
**Physicians**						
	**2-Way-Communication**		**Briefing**		**Feedback**	
**Year**	**t**_**0**_ **(2016)**	**t**_**1**_ **(2017)**	**t**_**0**_ **(2016)**	**t**_**1**_ **(2017)**	**t**_**0**_ **(2016)**	**t**_**1**_ **(2017)**
**Variables (β)**						
Supervisor Expectations	–	–	0.17	–	0.32***	0.25*
Teamwork Within Units	–	0.50***	0.31**	–	0.31**	0.52***
Psychological Safety	0.33**	–	0.40***	0.79***	0.26**	–
**Explained variance R**^**2**^	0.05**	0.20***	0.33***	0.44***	0.41***	0.48***
**N**	158	91	159	91	157	91

*Note:* Multiple regression analysis with calculated regression coefficient (β) and explained variance for all six models. Independent variables: *Supervisor Expectations*, *Teamwork Within Units*, *Psychological Safety*

Dependent variables: *2-Way-Communication*, *Briefing*, *Feedback*

Significance level: *p < 0.05 **p < 0.01 ***p < 0.001

Briefing: For nurses, all three safety culture aspects (*Supervisor Expectations* (β = 0.23, *p* < 0.001)*, Teamwork Within Units* (β = 0.33, p < 0.001)*, Psychological Safety* (β = 0.24, *p* < 0.01)) showed significant positive effects on *Briefing* at t_0._ At t_1,_ *Psychological Safety* (β = 0.41, *p* < 0.001) and *Supervisor Expectations* (β = 0.20, *p* < 0.05) had significant effects on *Briefing,* with *Psychological Safety* showing the strongest effect. The explained variance for the entire model decreased (R^2^ t_0_ = 23%; R^2^ t_1_ = 17%), resulting in a strong effect of f^2^ = 0.45. Concerning physicians, *Teamwork Within Units* (β = 0.31, *p* < 0.01) and *Psychological Safety* (β = 0.40, *p* < 0.001) were positively associated with *Briefing* at t_0_. However, at t_1_, only *Psychological Safety* showed a significant positive effect (β = 0.79, p < 0.001) on Briefing and considerably increased compared to t_0_. The explained variance for the entire model increased from R^2^ = 33% to R^2^ = 44% in t_1_, corresponding to a strong effect f^2^ = 0.89.

Feedback: For nurses, multiple regression at t_0_ showed again that all predictors ((*Supervisor Expectations* (β = 0.22, *p* < 0.001), *Teamwork Within Units* (β = 0.35, *p* < 0.001) and *Psychological Safety* (β = 0.33, *p* < 0.001)) were positively associated with *Feedback*. At t_1_, effects of 2 predictors reached statistical significance: *Supervisor Expectations* (β = 0.42, p < 0.001*)* and *Psychological Safety* (β = 0.53, p < 0.001). The explained variance for the entire model increased (R^2^ t_0_ = 34%; R^2^ t_1_ = 40%) resulting in a strong effect f^2^ = 0.82. For physicians, all 3 safety culture aspects showed significant effects on Feedback, while the regression coefficient for *Teamwork Within Units* increased to β = 0.52 (*p* < 0.001) at t_1_. In contrast, the positive effect of *Supervisor Expectations* decreased from β = 0.32 (p < 0.001) at t_0_, to β = 0.25 (*p* < 0.05) at t_1_. *Psychological Safety* showed no effect at t_1_. The explained variance increased to 48% at t_1_ (*p* < 0.01, f^2^ = 0.96).

## Discussion

Our results suggest that the interprofessional team-training for a small group of participants (9% of total staff in participating departments) resulted in changes in professionals’ perceptions with regard to communication practices. This may support a possibility for a successful transfer of training components into clinical practice by the means of champions. Nevertheless, the team training seemed to have more effect on communication practices than on aspects of safety culture. These findings are similar to those of Hefner et al., with the plausible explanation that team training addressed communication practices more likely than the influencing factors of supervisors and management [38]. A second explanation is provided by the study of Thomas and Galla, in which a change in aspects of safety culture presented itself much later than the change in communication practices [39]. One approach in order to solve this problem would be further training and data collection over a longer period of time.

The comparison of training participants and non-participants provided interesting results. When it comes to physicians, training participants had significantly higher scores compared to non-participants, which may provide evidence for the positive training effects. In contrast to this, no significant results could be observed when it comes to nurses. This leads to the assumption that the intervention had fewer effects on nurses than on physicians. One possible reason for this result could be different expectations and roles due to different professional backgrounds and hierarchical levels that already have been observed in other studies [40]. They may influence the perception of communication practices and safety culture aspects. For all physicians, the perception of communication practices showed significant changes at t_1_, indicating a successful transfer supported by the champions for this professional group. The significant differences between the participating and non-participating physicians suggest that the transfer could still be optimized. One opportunity to further strengthen the transfer could be to increase the number of trained champions.

The non-significant and at times negative changes we observed may be explained partially by response-shift bias [41], which occurs when the respondents’ understanding of the constructs in question improves between pre- and post-test, contributing to a more critical evaluation of practices than before and consequently lower scores.

We identified several differences in perceptions of nurses and physicians before and after the training. Physicians generally rated safety culture and communication aspects higher than nurses did, except for *Psychological Safety* after the training and *2-Way-Communication* at both points in time_._ These results are in line with previous studies [2, 42, 43]. Reasons for these differences may lie in different understandings of the underlying concept of safety culture and communication practices or in different management structures in nurses’ and physicians’ clinical work [42].

Regarding our second research question, descriptive results showed that physicians who already had higher values in safety-culture aspects compared to nurses before the training, had significantly higher values in two of three trained communication practices (*2-Way-Communication, Briefing*) after the training. This supports our theory that a high understanding of safety culture promotes the success of interprofessional team-training. Beyond these descriptive results, we identified *Psychological Safety* as the most important factor influencing all three communication practices for the nurses before and even stronger after the training. We found similar effects for physicians in *Briefing*_._ Results are comparable to a study by Tucker et al. [44], who identified psychological safety as an important aspect for implementing quality improvement practices. *Teamwork Within Units* was identified as the second most important factor, as we found very strong effects of teamwork on *Briefing to 2-Way Communication* for physicians after the training_,_ indicating a high relevance of teamwork for these communication practices. These results are related to previous findings, which suggest that culture is required as an important facilitator towards successful implementation of quality-improvement strategies [26].

In summary, our study has indicated that content of interprofessional training of champions can successfully be transferred into practice at the local level. Also, certain aspects of safety culture can promote this transfer of training content.

### Strengths and limitations

This study analysed changes in nurses’ and physicians’ perceptions of aspects of safety culture and communication practices using champions to transfer training contents into clinical practice. However, we found several limitations in the study. First, within this study, we used a pre-post design with one measurement before interprofessional team-trainings and the second measurement six months after completion of the trainings. Thus, our findings are limited to the two measurement points. In order to draw conclusions on long-term effects and sustainability of these interprofessional team-trainings, it would have been necessary conducting repetitive trainings and collecting further data (e.g. in combining a data collection on these specific topics with legally-required annual employee surveys) [45]. Second, due to data security requirements, data from participants of our pre- and post-surveys were not matched. Therefore, no other personal characteristics such as age or gender were collected. This limits in consequence our analysis to two separate evaluations of the two measurement points and less detailed analyses regarding personal characteristics. Thus, if possible, future studies should consider the possibility of matching data and conducting more detailed analyses on interaction effects regarding further personal characteristics. Third, we encountered a reduced response rate at the second measurement, a common problem in pre-post survey studies. Nevertheless, with about a 28% response rate at t_0_ and about 20% at t_1_, this study resulted in a sample size that is comparable to similar health services research studies, this was sufficient for the intended statistical analyses. Fourth, dependent and independent variables in the regression models were measured with the same survey, gathering subjective views of professionals and increasing the risk of common method variance bias [46].

Finally, with the complex structures and processes in a university hospital, it is possible that confounding variables remain undiscovered, but may have influenced the results.

## Conclusion

Results of this study suggest that interprofessional team-trainings of champions have a positive impact on routine clinical practice; as well they indicate the importance of safety culture aspects for successful transfer. For this, we recommend measuring the safety culture of the participating teams before starting an intervention. Future studies should address the question of how many champions are needed to achieve the greatest possible effect in the entire employee base.

## Supplementary Information

**Additional file 1: Table S1.** Used scales and their single item’s mean values and standard deviations (SD).

## Data Availability

Because of data security considerations, data from this study will not be made available in the public domain. However, data will be used by students of both project partners for their theses. Data will be stored in accordance with national and regional data security standards. Data are available from the last author upon reasonable request and with permission of University Hospital of Muenster.

## Abbreviations

GDPR: General Data Protection Regulation
HSPSC: Hospital Survey on Patient Safety Culture
SD: Standard deviation
