# Supplementary material for: Does interprofessional team-training affect nurses’ and physicians’ perceptions of safety culture and communication practices? Results of a pre-post survey study
Source: BMC Health Serv Res. 2021 Apr 14;21:341. doi: 10.1186/s12913-021-06137-5 (PMC8048288; doi:10.1186/s12913-021-06137-5)
Supplement: Supplementary file 1 — Additional file 1: Table S1. Used scales and their single item’s mean values and standard deviations (SD). [file 12913_2021_6137_MOESM1_ESM.docx]

| **Additional table 1.** **Used scales and their single item’s mean values and standard deviations (SD).** | | | | | | | | |
| --- | --- | --- | --- | --- | --- | --- | --- | --- |
|  | Nurses | | | | Physicians | | | |
|  | t0 | SD | t1 | SD | t0 | SD | t1 | SD |
| **Supervisor Expectations** | | | | | | | | |
| ﻿My supervisor/manager says a good word when he/she sees a job done according to established patient safety procedures. | 3.11 | 1.12 | 2.96 | 1.08 | 3.36 | 1.04 | 3.36 | 1.01 |
| ﻿My supervisor/manager seriously considers staff suggestions for improving patient safety. | 3.67 | 0.84 | 3.63 | 0.85 | 3.92 | 0.93 | 3.85 | 0.83 |
| ﻿Whenever pressure builds up, my supervisor/manager wants us to work faster, even if it means taking shortcuts. (R) | 2.79 | 1.00 | 2.69 | 0.99 | 2.82 | 0.94 | 3.00 | 0.99 |
| ﻿My supervisor/manager overlooks patient safety problems that happen over and over. (R) | 2.32 | 0.94 | 2.32 | 0.87 | 2.13 | 0.88 | 2.34 | 1.07 |
| **Teamwork Within Units** | | | | | | | | |
| ﻿People support one another in this unit | 3.71 | 0.69 | 3.64 | 0.71 | 4.06 | 0.74 | 3.95 | 0.86 |
| ﻿When a lot of work needs to be done quickly, we work together as a team to get the work done. | 3.74 | 0.76 | 3.67 | 0.82 | 3.91 | 0.82 | 3.78 | 0.93 |
| ﻿In this unit, people treat each other with respect. | 3.34 | 0.75 | 3.33 | 0.76 | 3.63 | 0.84 | 3.55 | 0.85 |
| ﻿When one area in this unit gets really busy, others help out. | 2.87 | 0.98 | 2.81 | 0.96 | 2.97 | 1.09 | 2.86 | 1.08 |
| **Psychological Safety** | | | | | | | | |
| ﻿If you make a mistake on this team, it is often held against you. | 2.29 | 0.99 | 2.04 | 0,92 | 2.15 | 0.92 | 2.12 | 0.90 |
| ﻿Members of this team are able to bring up problems and tough issues. | 3.40 | 0.96 | 3.47 | 0.89 | 3.59 | 1.01 | 3.63 | 0.95 |
| ﻿On our team everyone is accepted as they are. | 3.51 | 0.90 | 3.62 | 0.83 | 3.48 | 0.82 | 3.48 | 0.87 |
| ﻿It is safe to take a risk on this team. | 3.62 | 0.93 | 3.66 | 0.85 | 3.55 | 0.98 | 3.54 | 0.91 |
| ﻿It is difficult to ask other members of this team for help. | 2.20 | 0.90 | 2.14 | 0.90 | 2.02 | 0.73 | 2.13 | 0.89 |
| ﻿No one on this team would deliberately act in a way that undermines my efforts. | 3.72 | 0.97 | 3.79 | 0.97 | 3.59 | 1.05 | 3.65 | 1.00 |
| ﻿Working with members of this team, my unique skills and talents are valued and utilised. | 3.52 | 0.85 | 3.51 | 0.75 | 3.69 | 0.76 | 3.66 | 0.95 |
|  | Nurses | | | | Physicians | | | |
|  | t0 | SD | t1 | SD | t0 | SD | t1 | SD |
| **2-Way-Communication** | | | | | | | | |
| In the case of verbal orders (e.g. drug name and dose) we make sure by repetition that these have been understood correctly. | 3.75 | 0.96 | 3.69 | 0.96 | 3.17 | 1.08 | 3.43 | 0.33 |
| For us, it goes without saying that verbal orders are confirmed by repetition, especially in stressful situations (e.g. emergency situation). | 3.60 | 1.11 | 3.61 | 1.01 | 3.09 | 1.08 | 3.50 | 1.00 |
| Verbal repetitions of orders are taken seriously. | 3.71 | 1.06 | 3.67 | 1.01 | 3.31 | 1.08 | 3.65 | 0.99 |
| **Briefing** | | | | | | | | |
| It is important to us that all professionals involved in the care process have a common understanding of the situation and the planned treatment. | 3.61 | 0.87 | 3.74 | 0.82 | 3.71 | 0.91 | 3.86 | 0.78 |
| Before a shift begins, we coordinate our approaches with all professional groups involved in a structured manner. | 2.72 | 1.15 | 2.79 | 1.14 | 3.00 | 1.14 | 3.40 | 1.09 |
| Before an intervention begins, it is actively ensured that all participants have a common understanding of the situation and the procedure and that questions are clarified. | 3.13 | 1.01 | 3.30 | 0.98 | 3.66 | 1.05 | 3.96 | 0.85 |
| **Feedback** | | | | | | | | |
| We immediately discuss special situations (e.g. critical event, emergency situation) in the team with all professional groups involved. | 2.84 | 1.09 | 2.82 | 1.09 | 3.21 | 1.06 | 3.26 | 1.01 |
| In our clinic, it goes without saying that supervisors provide employees with feedback  on their actions. | 2.94 | 1.06 | 2.77 | 1.03 | 3.23 | 0.98 | 3.24 | 0.99 |
| In our clinic, it goes without saying that all professionals, regardless of hierarchy, can demand feedback on their actions. | 3.21 | 1.10 | 3.12 | 1.05 | 3.29 | 1.07 | 3.39 | 1.07 |
| Feedback takes place according to a defined structure. | 2.22 | 1.06 | 2.31 | 1.03 | 2.22 | 0.97 | 2.57 | 1.07 |
| Feedback is considered to be helpful and useful. | 3.42 | 0.93 | 3.34 | 1.01 | 3.42 | 0.87 | 3.65 | 0.86 |
